# Whole-Genome Shotgun (WGS) Sequence of *cis*-Isoprene Polymer-Degrading *Nocardia* sp. strain BSTN01

**DOI:** 10.1128/mra.01175-21

**Published:** 2022-03-14

**Authors:** Biraj Sarkar, Amit Kumar Mandal, Amit Ghati, Pranab Ghosh, Sukhendu Mandal, Ahmet Kati

**Affiliations:** a Laboratory of Molecular Bacteriology, Department of Microbiology, University of Calcutta, Kolkata, India; b Chemical Biology Laboratory, Department of Sericulture, Raiganj University, North Dinajpur, West Bengal, India; c Department of Microbiology, Barrackpore Rastraguru Surendranath College, Kolkata, India; d Department of Chemistry, University of North Bengal, Darjeeling, West Bengal, India; e Experimental Medicine Research and Application Center, University of Health Sciences Turkey, Uskudar, Istanbul, Turkey; f Department of Biotechnology, Institution of Health Sciences, University of Health Sciences Turkey, Uskudar, Istanbul, Turkey; Montana State University

## Abstract

Species belonging to the genus *Nocardia* are known to be facultative human pathogens. There are also reports of *Nocardia* species capable of degrading various forms of rubber. Here, we report the whole-genome shotgun (WGS) sequence of *Nocardia* sp. strain BSTN01, isolated from stored water in latex-collecting cups thrown away near a local rubber processing unit in Tripura, India.

## ANNOUNCEMENT

Species belonging to the genus *Nocardia* are aerobic, Gram-positive, nonmotile, filamentous actinomycetes and have been reported to be isolated from a wide range of environments (1–4). Numerous species belonging to the genus *Nocardia* have also been reported to be facultative intracellular human pathogens (1, 4–6). Studies have shown that some *Nocardia* spp. have the ability to degrade numerous toxic and harmful hydrocarbon-based materials. Different studies revealed that some *Nocardia* spp. harbor *lcp* genes, which are responsible for the initiation of the biodegradation of polymeric materials such as rubber (4, 7–10). The isolated strain BSTN01 was obtained from the stored water in latex-collecting cups (used to collect the freshly tapped latex from the Hevea brasiliensis trees) thrown away near a local rubber processing unit in Tripura, India. Initially, the collected water sample was serially diluted in sterile NaCl solution (0.9%), and 100 μL of the final dilution was spread on a sterile ISP-2 (International *Streptomyces* Project-2) agar plate (composition: malt extract, 10 g; yeast extract, 4 g; dextrose, 4 g; agar, 20 g, in 1 L of Milli-Q-grade autoclaved water, pH 7.0) supplemented with 50 μg/mL of both filter-sterilized cycloheximide and nystatin to check fungal contamination. The inoculated agar plate was then incubated for 4 to 5 days at 30°C. Thereafter, single colonies were individually picked and transferred to fresh ISP-2 plates using a sterile inoculating loop to obtain pure colonies using the streak plate method. The genome sequencing of the isolate *Nocardia* sp. strain BSTN01 was performed to explore the putative genes responsible for the rubber biodegradation and its possible pathogenicity (4).

**FIG 1:**
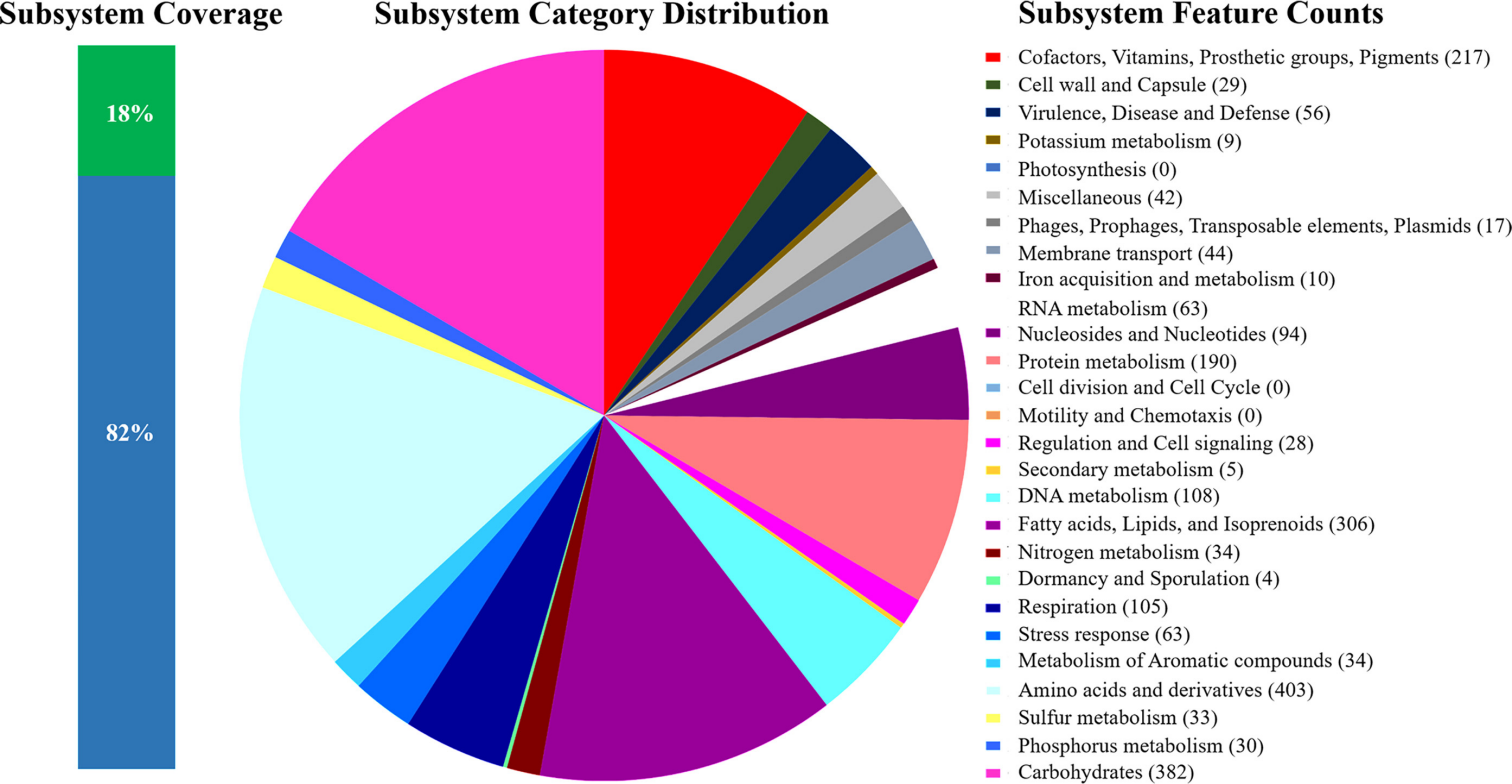
An overview of the subsystem categories assigned to the genome of *Nocardia* sp. BSTN01. The WGS sequence of the strain BSTN01 was annotated using the RAST server.

The strain BSTN01 was grown in ISP-2 broth medium (composition: malt extract, 10 g; yeast extract, 4 g; dextrose, 4 g, in 1 L of Milli-Q-grade autoclaved water, pH 7.0) at 30°C under shaking conditions at 400 rpm for 4 to 5 days. The genomic DNA was isolated from freshly grown cells of BSTN01 as per the standard phenol:chloroform method (11, 12). The paired-end libraries were prepared and sequenced using the Illumina HiSeq X10 platform (AgriGenome Labs Pvt. Ltd., Kochi, Kerala, India), producing a total of 14,888,026 reads with 2 × 150-bp paired-end read length. The DNA library was prepared using the NEBNext Ultra DNA library prep kit according to the manufacturer’s manual. To accomplish data preprocessing, unique reads were first fetched using BBTools v38.57 (https://sourceforge.net/projects/bbmap) (13). Adapter removal and low-quality end trimming was done using the Adapter Removal-v2 tool v2.3.1 (https://github.com/MikkelSchubert/adapterremoval) (14). The preprocessed reads were aligned with a plasmid sequence database (in-house database curated from NCBI RefSeq by AgriGenome Labs Pvt. Ltd.), and the unaligned reads were used for performing assembly. The *de novo* assembly was completed using the Unicycler v0.4.8 (https://github.com/rrwick/Unicycler) assembler (15). The annotation was carried out employing the NCBI Prokaryotic Genome Annotation Pipeline (PGAP) v4.13 with the methods “best-placed reference protein set” and “GeneMarkS-2+” (16). The assembly produced a draft genome sequence encompassing 208 contigs. The *N*_50_ length is 108,911 bp, and the *L*_50_ count is 20. The estimated genome size is 7,795,358 bp with a 67.7% G+C content and 96.3× coverage. A total of 7,253 coding sequences were annotated, including 6 rRNA genes (2 16S and 4 23S) and 49 tRNAs. The WGS sequence of BSTN01 was also later uploaded for rapid annotations in the Rapid Annotations using Subsystems Technology (RAST) server (17). For all the software tools used, default parameters were applied unless otherwise specified. The total number of coding sequences predicted was 7,702, and the number of predicted RNAs was 51. An overview of the subsystem categories assigned to the genome of *Nocardia* sp. BSTN01 is shown in Fig. 1. The WGS analysis revealed the presence of the *lcp* gene (responsible for the initiation of the biodegradation process of rubber) along with other virulence genes (e.g., *mce*, *SodA*, *KatG*, *ahpC*, etc.) (4). Further insight into the genome of BSTN01 will undoubtedly contribute toward the molecular basis of rubber biodegradation for solid-polymeric-rubber waste management.

### Data availability.

This whole-genome shotgun project has been deposited at NCBI under the accession number JADKYP000000000. The version described in this paper is the first version, JADKYP010000000. The BioSample and BioProject accession numbers are SAMN16604275 and PRJNA673320, respectively. The raw data are available from the Sequence Read Archive (SRA) under the accession number SRR17194475.
